# The Effect of Tree Spacing on Yields of Alley Cropping Systems—A Case Study from Hungary

**DOI:** 10.3390/plants12030595

**Published:** 2023-01-29

**Authors:** Veronika Honfy, Zoltán Pödör, Zsolt Keserű, János Rásó, Tamás Ábri, Attila Borovics

**Affiliations:** 1 Department of Plantation Forestry, Forest Research Institute, University of Sopron, Farkassziget 3, H-4150 Püspökladány, Hungary; 2 Faculty of Informatics, Eötvös Loránd University, Pázmány Péter str. 1/C, H-1117 Budapest, Hungary; 3 Department of Tree Breeding, Forest Research Institute, University of Sopron, Várkerület 30/A, H-9400 Sárvár, Hungary

**Keywords:** temperate agroforestry, silvoarable, cereal, spatial arrangement of trees, dendromass, land equivalent ratio

## Abstract

Alley cropping is a specific agroforestry system, which is regarded as sustainable land use management, that could play a crucial role in climate change adaptation and mitigation. Despite its appealing attributes, farmers’ up-take of the system is slow in temperate regions. This study aims to contribute to scaling-up agroforestry through a case study in Hungary and to help to design productive alley cropping systems. We investigated which tree planting pattern of black locust (*Robinia pseudoacacia* L.) results in the most productive alley cropping system when intercropped with triticale (x *Triticosecale* W.) by statistically analysing the yields of the intercrop and of the trees in nine different layouts and by calculating land equivalent ratios (LER). There was significant difference between the treatments both in triticale and black locust yields. The more trees planted on a hectare, the higher the volume of the stand, and the less yield of triticale was observed, although the latter correlation was weak and in some cases the triticale was more productive between the trees compared with sole crop control. Eight out of nine treatments had favourable LER (0.94–1.35) when the trees were five years old. Black locust and triticale seem to be a good combination for productive alley cropping systems.

## 1. Introduction

The main objective of this study is to provide data on yields of innovative alley cropping systems to contribute to scaling up agroforestry in Hungary and in temperate regions. Agroforestry systems—such as alley cropping—provide economic, social and environmental benefits as compared with conventional farming systems. Relevance of agroforestry research becomes tangible when considering that more than 40% of the world’s agricultural land has over 10% tree cover [1]. Agroforestry is defined as a sustainable land use practice, where woody perennials are purposely grown with other crops and/or are combined with livestock or with other agricultural production, in some form of spatial arrangement or temporal sequence for economic and/or ecological benefits. Alley cropping is a specific type of agroforestry/intercropping system combining fruit, nut, or high-value timber production with crop production in between the tree rows, but it can also be combined with livestock. Trees provide shelter to crops and animals and shade against the sun, the hail and the wind. In the meantime, competition for resources may take place, such as for the nutrients, water and light, which can result in a substantial decrease in the yields, as is often recorded in intercropping and in some research conducted on alley cropping systems [2,3,4]. From the point of forestry, non-stand-forming tree species which bare valuable wood or fruits can play a role [5]. Many environmental challenges can be addressed by planting trees into the farm or landscape, as they protect soil and water, improve nutrient cycles and microclimate and they enhance the carbon sequestration potential of agricultural fields. Depending on the choice of tree species there is potential for apiculture, too. Through the diversification of a farm, not only biodiversity is enhanced, but timber, fruits and nuts can be alternative sources of income [6,7]. When trees utilise resources which are otherwise unavailable for the adjacent crops or vice versa, the productivity of agroforestry systems can be higher than that of conventional crop production or forestry. Examples are water-availability in deeper layers of the soils, which can only be reached by the roots of trees, or when young forest plantations are not able to fully utilise solar radiation, which is then complemented by the crops. Trees also play an important role in agroecosystems from the point of crop protection, as they provide habitats for natural enemies, which then move on to field plants [8,9]. Well-designed agroforestry systems tend to have favourable land equivalent ratios (LER), and these complementary processes—when companion crops are chosen adequately—also explain potential productivity and profitability. Because the life cycle of trees is much longer than that of agricultural crops, developing models are crucial to predict productions and economic outcomes in the long run, which is a complex task [10,11,12].

Regardless of the many favourable attributes of agroforestry systems, farmers’ adoption of it is very slow, specifically in temperate regions (such as in Europe) due to several reasons, such as the complexity of the systems, lack of knowledge available, lack or inadequate incentives, which are key elements in agroforestry where trees are regarded as long-term investments [13]. Depending on the tree species and on the goal of the tree plantation it may be 2–4 years for energy plantation, even more years for some fruit trees and up to 12–30 years in the case of fast-growing timber species until financial return is applicable. To be able to calculate economic return more studies are required on the productivity of alley cropping systems [14]. On the other hand, fruit growers may implement intercropping between fruit trees for annual income before the trees start to produce fruits or nuts [15]. Whatever the case may be, one must be careful when designing and implementing such systems, as there are several complex ecological processes that occur which have to be understood, to make sure that ecosystems are rather improved and not damaged [16]. There are several studies which extensively investigated ecological and environmental interactions, trade-offs and benefits, and agroforestry’s role in climate change adaptation and mitigation [17,18]. Few studies have actually looked at the yields specifically in different tree planting patterns [19,20,21], while such studies are crucial for the uptake of alley cropping systems by the farmers.

The aim of this study is to determine which tree planting patterns provide the most beneficial alley cropping design by the means of production. This work shall be regarded as a foundation of alley cropping research in Hungary, specifically as this is the first one of its kind where cereal production is combined with widely spaced trees for timber production, as of the best of the knowledge of the authors. In this field experiment, different tree planting schemes served as treatments on triticale and back locust production, and eventually the overall yield of alley cropping systems were determined and the land equivalent ratio was calculated. Black locust is a multifunctional nitrogen-fixing tree species which is of great international interest; alongside triticale, both species play an important role in the changing climate, with their drought-tolerant attributes and due to their potential in marginal sites and their resistance against pests and diseases. Results can be relevant for agroforestry research worldwide, but mainly in temperate climates, as such studies are particularly scarce [15,22,23]. Furthermore, the results could serve as valuable inputs for biogeophysical and economical modelling, as well as for calculating climate change mitigation potential.

## 2. Results

### 2.1. Features Defining each Treatment

The key parameters that defined each treatment were calculated based on the tree spacings and are summarised in Table 1. All this information was needed to be able to finally determine the area which was available for sowing. Applying a 3-m strip for each tree row (in all treatments), 70% of a hectare was sown in the case of 9-m row space, while in the case of 15 and 21 m row space 81% and 86% of a hectare was utilised by the intercrop, respectively.

### 2.2. Tree Component

#### 2.2.1. Above-Ground Dendromass of Black Locust

Based on dendrometric measures, the average tree volume and mass (absolute dry matter) was determined following the vegetative period of 2018 and 2019. The trees were 4 and 5 years old in this period (Table 2).

In 2018, the average above-ground dry dendromass of trees varied between 12.0 and 18.0 kg at the age of four. The highest values were recorded in the treatment 15 × 2, 15 × 3 and 9 × 2, where the growing spaces were 30, 45 and 18 m^2^, respectively. The lowest value was observed in 9 × 1, in the densest plantation. As the result of the Kruskal–Wallis test (α = 0.05)—based on 272 samples—there was a significant difference between the treatments. Treatments 15 × 2 and 15 × 3 (the highest values) were significantly higher than 9 × 1. Treatment 15 × 2 was also significantly higher than 21 × 2. There was no significant (level α = 0.05) difference between the rest of the treatments.

In 2019, when the trees were 5 years old, their above-ground dry dendromass varied between 16.5 and 27.0 kg. The highest values were obtained at treatments 15 × 3, 15 × 2 and 9 × 2, similarly to the previous year, with 27.0, 26.2 and 21.0 kg, respectively. Based on 263 samples, the pairwise comparisons, following the Kruskal–Wallis test (α = 0.05), showed that (Table 2) at treatments 15 × 3 and 15 × 2 the dry mass was significantly higher than that in the 9 × 1 and 21 × 2 layouts, and in addition—compared with the previous year—was also significantly higher than 21 × 3. The rest of the treatments did not differ significantly.

**Figure 1:**
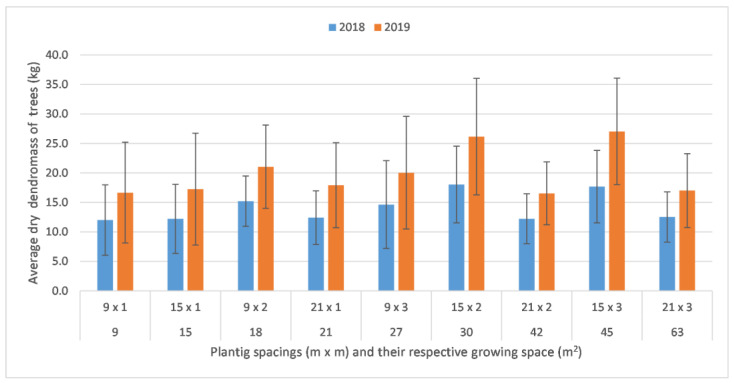
Average above-ground dry mass of black locust trees according to different tree spacings in agroforestry systems at the ages of 4 and 5. The growing spaces are indicated below the tree spacings. The error bars denote standard deviation (α = 0.05; 2018: n = 272; 2019: n = 263).

To summarise the result of the two years, individual black locust trees gained the highest average above-ground dry mass (therefore the highest average volume) at the growing spaces 18, 27 and 30 m^2^ (Figure 1). The results of 30 and 45 m^2^ were significantly higher (α = 0.05) than those of treatment 9 m^2^ in both years. At the age of five, additional significant differences appeared compared with the best performing treatments (30 and 45 m^2^), namely at the growing spaces 42 and 63 (21 × 2 and 21 × 3). The most and the least dense stands were less productive than the rest of the treatments, as a result of the two investigated years.

The total volume and total above-ground dry dendromass of stands per hectare at the ages of 4 and 5 are in Table 3.

**Figure 2:**
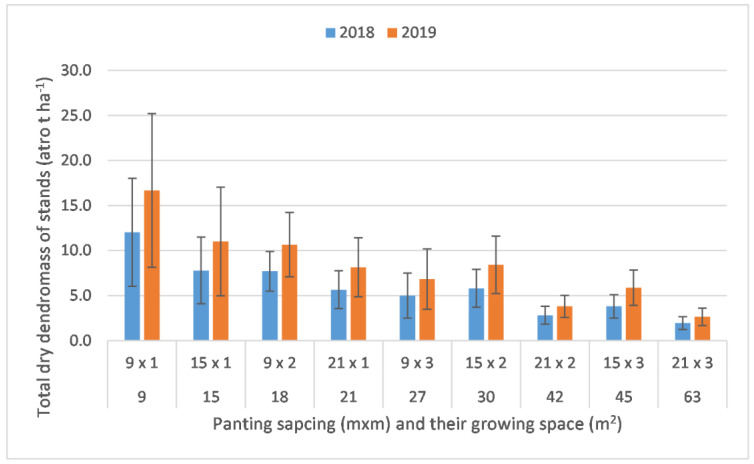
Total above-ground dry dendromass of agroforestry stands per hectare according to different tree spacings (α = 0.05; 2018: n = 272; 2019: n = 263).

Planting spacings determined the number of trees in each treatment, which at the age of five resulted in dry dendromass values between 2.6 and 16.7 atro t ha^−1^ (3.6–22.9 m^3^ ha^−1^), which demonstrates over six-fold of above-ground dendromass production in the case of the densest stand compared with the least dense layout. The higher the growing space the lesser the total dendromass, except for treatment 15 × 2 (30 m^2^) at the age of four and treatments 15 × 2 and 15 × 3 (30 and 45 m^2^, respectively) at the age of five; these layouts also had the highest production when analysed on the basis of yield of individual trees (Figure 2). Based on the results of Kruskal–Wallis test (α = 0.05), the pair-wise comparison showed significant difference between the treatments, which are shown in Table 3. While 9 × 1 was significantly higher than most of the treatments, there was barely any significant difference between the treatments (except for one case) where the growing space varied between 15 and 30 m. Regarding the lowest values of total dry dendromass per hectare, the yields at growing spaces of 42 and 63 m^2^ tended to be significantly lower than the rest of the treatments. This is probably because the bigger growing spaces cannot compensate for the overall lower number of trees on a hectare basis. The only exception was treatment 15 × 3 (45 m^2^), which did not differ significantly from most of the treatments, and it is possible that the row spacing of 15 m may have had a positive effect on the growth of the black locust trees at the ages of 4 and 5, but this shall be investigated further. The findings are very similar in the two investigated years.

#### 2.2.2. Relationship between the Growing Space and Total Volume of the Stands

**Figure 3:**
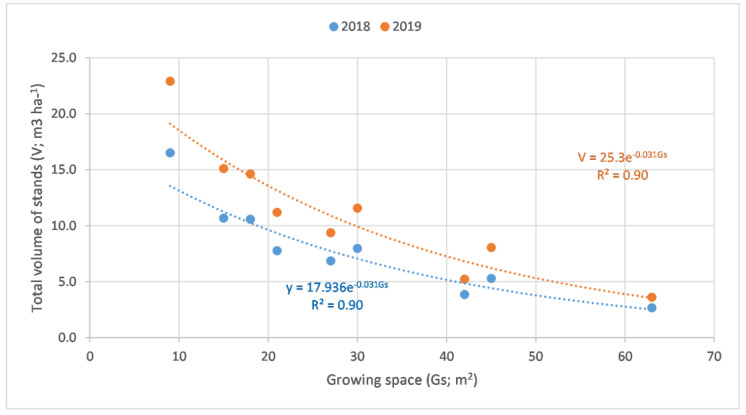
Relationship between the total volume of stands and the growing space per hectare in alley cropping systems at the ages of 4 and 5 (α = 0.01, n = 9).

There is a strong correlation between the total volume of the stand and the growing space (Figure 3). Smaller growing spaces mean that there are more trees present in a given area. The correlation between these two parameters can be described with simple linear regression, using a significance level α = 0.05, where the deterministic variables R^2^ values at the ages of 4 and 5 are 0.90 in both of the years.

### 2.3. Crop Component

#### 2.3.1. Yields of Triticale

The mean dry matter (DM) yields of triticale on a m^2^ basis and ton per hectare are shown in Table 4 for the harvest years of 2018 and 2019, when the trees were 4 and 5 years old, respectively. The statistical analyses on a m^2^ basis were only completed for the treatments with the same sowing rate in each year.

In 2018, the average yield varied between 280.9 and 425.8 g m^−2^, therefore in some cases the production was about 1.5 times higher in some treatments compared with each other. The lowest yield was recorded in treatment 21 × 3 and the highest in 15 × 2. The control area (without trees) yielded 335 g m^−2^, which is similar to the value of treatment 15 × 3, and it was exceeded in three other treatments, where trees were present. The highest yields in descending order are as follows: 426 g m^−2^ in treatment 15 × 2, 392 and 352 g m^−2^ in treatments 21 × 2 and 21 × 1, respectively. The results of the Kruskal–Wallis test revealed that there was a significant difference between the treatments (α = 0.05). The results of pairwise comparisons are indicated in Table 4. At treatment 15 × 2, yield was significantly higher than almost all of the treatments—except for 21 × 2—but including the control. Regarding the control, the only significant difference was the above-mentioned case—15 × 2, where excess yield was recorded—but it did not differ significantly from any other treatment, so there was no significantly less productive treatment.

To summarise this for the year 2018, on a m^2^ basis there was no treatment which was significantly lower compared to the conventional way of cereal production (without trees); indeed, in several cases the yields between the trees were similar or even higher.

Looking at the yields on the basis of per hectare, in the case of the treatments of 9 m row spacing (9 × 1, 9 × 2 and 9 × 3)—where the sowing rate was higher—yields varied between 1.90 and 2.85 tons, whereas at the rest of the treatments it varied between 2.40 and 3.44 t ha^−1^. The lowest yield was acquired at treatment 15 × 1, while the highest was at treatment 15 × 2, and it has to be noted that the difference is fairly high, being 1 t ha^−1^. It has to be highlighted that these results apply to different sizes of land units. Although the presence of trees reduced the sowing area, triticale had similar yields in several cases in the alley cropping systems compared to the control treatment. In regard to this, triticale was highly productive in treatments 15 × 2 and 21 × 2, where only 85% and 81% of one hectare was sown, respectively, yet the yield was nearly the same as in the control, where 100% was sown. It can also be noted that when looking at the treatments which belong to the same row spaces, in most cases the 2-m in-row space had the highest yields.

In 2019, the average yield per m^2^ varied between 110.9 and 262.1 g, and in some cases the difference among the treatments was over two-fold. In this year, the treatments of 9 m row space (9 × 1, 9 × 2 and 9 × 3) were also included in the statistical analysis. The lowest yield was recorded at 9 × 3, and the highest—similarly to the previous year—at 15 × 2. The control yielded 192.6 g m^−2^. Similar to this, but somewhat lower, was the production of 21 × 2. All of the treatments amongst 9 m row space had lower yields than the control. There was a significant difference (α = 0.05) between the treatments based on the result of the Kruskal–Wallis test. The outcome of the pairwise comparisons is shown in Table 4.

The highest yield was observed—similarly to the previous year—at treatment 15 × 2, which did not differ significantly from treatments 15 × 3, 21 × 1 and the control, but it was significantly higher than the rest of the treatments. Treatment 9 × 2 was fairly productive and it was only significantly lower than treatments 15 × 2 and 21 × 1. It is worth mentioning treatment 9 × 3, which was the least productive treatment, being significantly weaker than the rest of them. Comparing the results to the control treatment, triticale only had significantly (level α = 0.05) lower yield between the trees at the above-mentioned treatment 9 × 3, but there was no significant difference to the rest of the layouts when analysing data on a m^2^ basis.

To summarise the results in 2019, in several cases the yield of triticale on a m^2^ basis did exceed the yield at the control area, but unlike in the previous year, there was no significantly higher yield recorded between the trees.

On a hectare basis yields varied between 0.78 and 2.01 t ha^−1^ in the alley cropping systems, while 1.93 t ha^−1^ was harvested from the control plot. The lowest yield was observed at treatment 9 × 3 and the highest at 15 × 2, but again it has to be underlined that the sowing areas vary depending on the row spacings. Similarly to the previous year, in three cases—treatments 15 × 2, 15 × 3 and 21 × 1—the yields were very close to that in the control, although those were acquired in 14–19% smaller sowing areas.

#### 2.3.2. Relationship between the Number of Trees and the Yield of Intercrop

**Figure 4:**
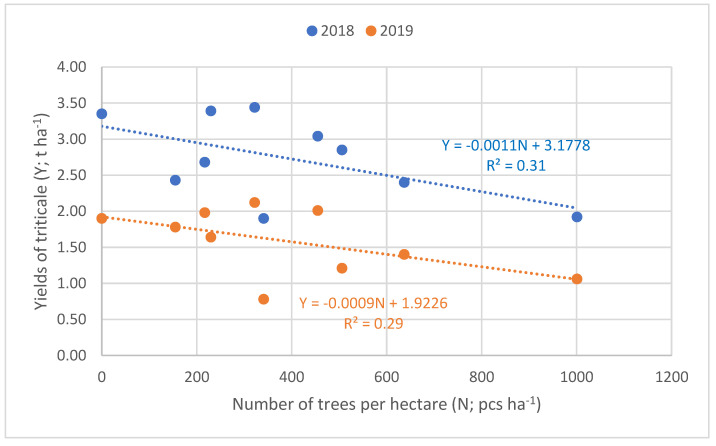
Relationship between the yields of the intercrop in alley cropping systems with different number of trees per hectare (α = 0.05, n = 9).

The correlation between the yield of triticale and the number of black locust trees per hectare was described using simple linear regression (Figure 4) with a significance level α = 0.05, where the deterministic variables R^2^ values were 0.31 and 0.29 when the trees were 4 and 5 years old, respectively. There was a negative relationship between these two parameters. The more trees there were the less yield was observed.

### 2.4. Yields of the Two Components Combined in an Alley Cropping System

**Figure 5:**
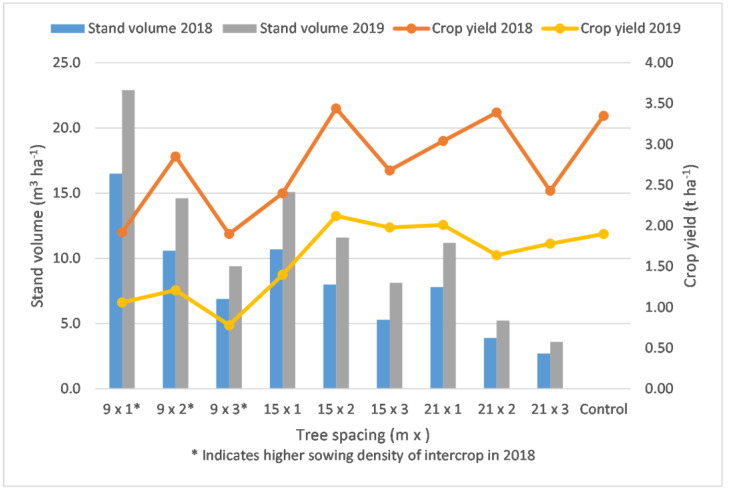
Yields of triticale–black locust alley cropping systems according to the tree spacing in 2018 and 2019, when the trees were 4 and 5 years old, based on the stand volume.

**Figure A1:**
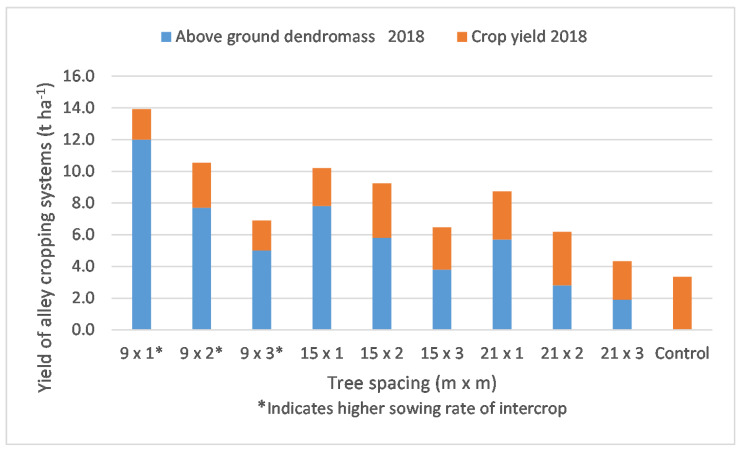
Yields of triticale–black locust alley cropping systems in 2018 according to the tree spacing, based on above-ground dendromass. The trees were 4 years old.

**Figure A2:**
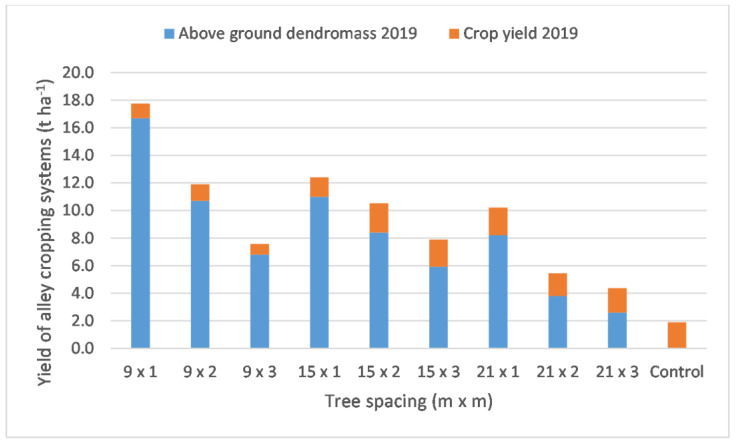
Yields of triticale–black locust alley cropping systems in 2019 according to the tree spacing, based on above-ground dendromass. The trees were 5 years old.

The overall production of the alley cropping systems according to the tree spacing is summarised in Figure 5 by presenting the volume of the stands and the mass of the agricultural crop of each treatment in the two investigated years. The highest yields of triticale per hectare were recorded in treatment 15 × 2 (322 trees per hectare) in both of the years, while the highest above-ground dry dendromass/volume was obtained at the densest stands, 9 × 1 (1001 trees per hectare), for both years. The lowest yields of triticale were observed in the treatments of the nine-metre row space for both years, where only 81% of the land was utilised by the crop. Regarding the control, there were cases of lower and higher yields in both years, although in every treatment the sowing area was reduced by 14–30%. In addition, the yields of the trees add to the overall production in the alley cropping systems. To be able to visualise the results, and to tell which treatments were the most productive, a sum of the crop yield and the above-ground dendromass was calculated on a ton per hectare basis for each investigated year, which are presented in Appendix A (Figure A1, Figure A2). 

### 2.5. Land Equivalent Ratio

The results of the LER were based on total volume per hectare of black locust stands, and are shown in Table 5. 

The table is ordered by descendent manner of LER values of the agroforestry systems, which varied between 0.64 and 1.35. Altogether the results are favourable, as eight out of nine treatments are nearly equal to or above one. Only treatment 9 × 3 was clearly way below one, with LER 0.64. Treatment 15 × 2 ranked the highest with LER 1.35 followed by 21 × 1 and 15 × 3 with LER 1.29 and 1.19, respectively, while 9 × 1 and 15 × 1 had the same LER value of 1.07.

## 3. Discussion

### 3.1. Tree Component

While Ivezić et al. [14] collected and analysed the results of relevant research papers regarding crop yields in temperate alley cropping systems, no such compendium is available regarding the yield of tree components. Rather, recording height, diameter and measuring biomass production in short rotation coppice is more common in studies where the tree component is investigated [20,24]. Another case is when fruit trees are grown in the alley cropping system, and thus no data on timber is recorded. In research conducted in Croatia [15], maize, barley and rapeseed were grown between allies of walnut trees with different tree planting densities per hectare (170, 135 and 100 trees ha^−1^), using the Farm-SAFE and Yield-SAFE models. However, the analyses focused on walnut fruit and not on the timber production. Often, the yield predictions are based on modelling, while in some cases they are validated, such as the case in the above-mentioned study, where yields for some years were measured. 

While forestry works with planting spacings and growing spacing, analysing an alley cropping system requires evaluation on a tree per hectare basis, which does not specify the row and in-row spacing which is a determinant factor for the growth of trees. The yield of individual black locust trees significantly differed in both investigated years depending on the tree spacing. The highest yields per individual tree were recorded in treatment 15 × 2, with 30 m^2^ growing space; treatment 15 × 3 yielded similar, where growing space was 45 m^2^. Dupraz et al. [25], in the project called Silvoarable Agroforestry for Europe (SAFE), determined reference yields on the basis of individual tree volume to be able to compare yields in forestry and agroforestry management. According to the yield table of black locusts in Hungary, at the age of five a stand consisting of 4045 trees would yield 45 m^3^, which means 0.011 m^3^ [26] per tree, in contrast to our results ranging between 0.023 and 0.036 at the same age. Because trees in agroforestry stands are spaced widely, less competition occurs between them in the beginning, which can explain this result. In the meantime, too large spacing between tree rows (such as 21 m) at this age may be unfavourable for tree growth, as competition for light actually contributes to higher tree heights, which could be the explanation for the best performance in 15 m row spacing treatments at the ages of four and five. These findings are expected to change with time, and best performance could be maintained with properly planned thinning of the stands.

As in forestry, there was a strong relationship between the growing space and total volume of the stands. Khan and Chaudhry [21] did report the same observation in an 8-year-long study, where a poplar variety was intercropped with alternate wheat and fodder maize and, similarly, the aim of the study was to investigate the effect of spacing and planting density on tree growth at 455 (3.7 × 6.1), 305 (3.7 × 9.1) and 230 (3.7 × 12.1) trees per hectare. There was significant difference (α = 0.05) between the yields on a hectare basis, where 1001 trees per hectare had the highest above-ground dendromass and 155 trees per hectare resulted in the lowest yields, yet significant difference was barely found in treatments where the number of trees per hectare varied between 322 and 637. Planting spacing does have an effect on the yields of the stands, but it needs further investigation to see whether the row spacing or in-row spacing is more determinant. It is difficult to compare the findings with other authors’ results, as tree species, age, site and densities are greatly varied in those few studies found in this topic. 

### 3.2. Crop Component

Because in any case the area available for cropping will always be reduced in an alley cropping system, due to the trees taking up some space, comparing the yields on a square metre basis can show the actual effect of the trees (so we excluded the effect of reduced sowing space as it is the case when looking at yields on the basis of yield per hectare). This way, there was no significantly (α = 0.05) lower yield found in any of the tree planting patterns compared to sole crop production, yet in several cases the yields between the trees were similar, or even significantly higher (15 × 2 and 21 × 2) when the trees were 4 years old. This could be explained by the nitrogen-fixing attribute of black locust trees, of which the intercrops may benefit. As the presence of trees can enhance nutrient cycles, it can also improve microclimate and contribute to more favourable water regimes in certain weather conditions, which may result in higher yields of triticale compared with sole cropping. As trees provide habitats for natural enemies of pests, plants are likely to be healthier and thrive if such natural predators are present and biological control takes place. In a study conducted in Canada [27] it was found that new generations of alley cropping systems where trees are planted with wide row spacing (25–90 m) are more adapted to large-scale annual crops (maize, soybean, wheat and forage crops), as trees had neutral effects on the intercrops, and that competition for water between the crops and the trees was neglectable. One year later we did not find significantly higher yields of the intercrop on a square metre basis anymore, but it was still higher at the following tree planting patterns: 15 × 2, 15 × 3, 21 × 1 and 21 × 3. Thus, it was only true for the wider row spacings (>9 m). This is most likely due to enhanced competition between trees and crops for the resources, as trees grow with age [15].

Some of the planting patterns did not decrease triticale production per hectare. When the trees were 4 years old, the crops between 15 × 2 and 21 × 2 tree spacings and the sole crop production all resulted around 3.4 t ha^−1^, although only 81% and 86% of the total land area was sown when 322 and 230 trees were grown, respectively. When the trees were 5 years old, applying 15 × 2 (322 trees ha^−1^), 15 × 3 (217 trees ha^−1^) and 21 × 1 (455 trees ha^−1^) planting patterns resulted in a similar yield to the sole crop control. These findings are in line with the results of a meta-analysis conducted by Ivezić et al. [14], where, after looking at 13 studies on crop production in temperate alley cropping systems, they found that in the year of tree planting, the relative yield in the tree alleys were 96% of the sole crops. In our case it was also valid, and it was actually above 100% for several planting densities when the trees were 4 and 5 years old, while the above-mentioned study predicted a decline of 2.6% yield loss in each coming year. 

To summarise our findings on the yield of intercrop, the presence of trees did not decrease the yields compared to the control on a square metre basis. Thus, lower triticale yields on a hectare in between 4 and 5 year old black locust trees shall rather be explained by other factors, such as reduced cropping area available, or it could also have been affected by the soil, the microclimate or due to biodiversity aspects. There are many above- and below-ground interactions in the soil–plant–tree nexus [16] that could also contribute to higher yields, as was also observed by Seserman et al. [28]. Additionally, Burgess et al. [29] found that in cases when the intercrop is in a well-developed stage by the time the trees develop their crown (cereal–poplar combination) the decrease in yield near the trees is not significant. In Italy, a similar conclusion was made in the case of walnut and clover [25], and it was actually the case in this study, where a winter variety of triticale was grown with black locust, which flowers and starts to leaf around the end of April or beginning of May in Hungary.

It is possible that black locust trees even contributed to higher crop yields in some cases. When looking at the treatments which belong to the same row spaces, most of the cases in the 2-m in-row space had the highest yields in a hectare of agroforestry, which suggests that adequate tree spacing could also favour these positive processes. As black locust trees are a nitrogen-fixing tree species, plants could have benefited from this capability of the tree, which would be worth investigating. As to the shade effect, a study [22] found that when a barrier was applied between the roots of trees and maize there was no decline in the yield of maize adjacent to the tree, but it yielded as much as the control plot without trees, suggesting that the shading did not explain changes of yields of intercrop close to the trees, but rather below-ground interaction(s) that occurred. On the contrary, in an experimental site in France, pruning the roots of poplar had no effect on the intercropped cereal, yet when the effect of shading was investigated by pruning the branches, significant differences were found [25]. All this highlights the importance of good companion crops when planning an agroforestry plot to minimise yield loss.

### 3.3. Evaluating the Alley Cropping Systems and LER

Yields of triticale decreased with an increase in tree density. This was explained by a linear regression model where the coefficient of determination values were R^2^ = 0.29 and 0.31 in the two investigated years, yet there was a significant relationship (α = 0.05), so the yield loss per hectare of the crop can partly be explained by the number of trees per hectare. Ivezić et al. [14] found similar results in the meta-analysis of yields in temperate alley cropping systems. Seiter [20] also found that a high ratio of tree occupation was negatively correlated with com yield. It has to be highlighted that it is not only the number of trees, but perhaps the planting pattern that plays a role, as the wilder the rows between the trees, the more area is available for cropping. For example, applying a 3-m strip for each tree row (as was the case in our study), 64% of a hectare was sown in the case of 9-m row space, while in the case of 15 and 21 m row spaces 79% and 85% of a hectare was utilised by the intercrop, respectively. Additionally, reducing the unsown area between the trees and crops can add to the ratio being cropped.

Altogether, the land equivalent ratios found in this study were appealing, as eight out of nine planting patterns were nearly equal to or above one. The favourable values varied between 1.07 and 1.35, while further three planting patterns resulted in LER 0.94 to 0.99, which is still worth considering in regard to other ecosystem services which trees could provide on a farm. The finding of Ivezić et al. [14], which was that every extra 100 trees would cause a 20% decrease in the crops’ yield, was not the case in our predicted triticale–black locust agroforestry LER, although it is only a case of one year, at one location, when the trees were 5 years old. Nevertheless, it does draw attention to the idea that tree density may not be precise enough to withdraw far-reaching conclusions of LER, as different tree planting patterns may change the overall yield of the system. This could be investigated with the same amount of trees per hectare, but with different planting spacings. 

In three cases we found that LER values of triticale were between 1.01 and 1.08 (at planting spacing 15 × 2, 21 × 1 and 15 × 3), which already contributed to a positive LER value by themselves. These were also the most productive cases when looking at the yields on a square metre basis, so even though the ratio of sown area was lower, the higher yields compensated the ’field loss’ on a hectare basis. Likewise, Žalac et al. [15] also reported high relative crop yields in a grain maize–barley–rapeseed rotation in a walnut alley cropping system, with LER values 1.05–1.14 in years 1, 4 and 7, predicted by the Yield-SAFE model. In that study, a simulation of maintaining the system for 20 years resulted in LER 1.38–1.53 depending on the number of trees per hectare (170 and 100 trees, respectively). The first 5 years of crop yields were validated with high accuracy. Graves et al. [30] also found that, in the long term, LER will decrease more in the case of higher tree densities compared with systems with fewer trees; when running the model with 50 and 113 trees per hectare, the results were 1–1.4 LER values, respectively. While the LER values predicted in our study do fit in with the findings of literature, it has to be investigated in the long term. 

The aim of this study was to determine which tree planting patterns provided the most beneficial alley cropping design by the means of production. According to LER, we found that 15 × 2 (322 trees) was the most productive version of the tree planting patterns of the trial at the age of five of the black locust trees, which was also the most productive version regarding triticale production (in both of the years). Considering wood production, the densest tree planting patterns yielded the highest at tree spacing 9 × 1 (1001 trees), although if the main objective of the alley cropping system is wood production, much higher tree densities are suggested.

## 4. Conclusions

The study presented here is a foundational work of alley cropping systems in Hungary, where a demonstration site was established and field research has begun. Therefore, the current results are only applicable for the investigated years and site, with the species (and variety) tested, and we cannot draw far-reaching conclusions from the findings. Nonetheless, the combination of black locust and triticale seems to be a good choice with the wider row spacing (>9 m), and by extending the investigations in time and space, more general results can be available in the future. Our findings are expected to change with time as the trees grow, and best performance of the alley cropping system shall be maintained with properly planned thinning and pruning to manage tree–crop competition, as long as intercropping is regarded beneficial to the farmer financially, or due to other ecosystem services. Therefore, to decide which treatment could be the best choice in a farming context, one must define the main purpose of the alley cropping system (which may also change through the system’s development), such as crop production, timber or biomass production or other purposes (biodiversity aspects, habitat, soil protection, etc.). One relevant aspect could be accounting for carbon sequestration, where trees and the soil beneath them are considered carbon pools for the long term, which may also be rewarded in the carbon market.

## 5. Materials and Methods

### 5.1. The Experimental Site

The alley cropping experiment was created in 2016–2017 by the former research centre NARIC (National Agricultural Research and Innovation Centre) as a cooperation between its institutions, namely: the Forest Research Institute and the Institution of Agricultural Engineering. The experimental site is located in Gödöllő, Hungary (47°35′25.4″ N 19°19′55.0″ E) in the undulating area of Gödöllő Hills. This region can be described as a transitional zone between the bottom of the hills of the North Hungarian Mountains and the edge of the Hungarian Great Plain, and its geology is characterised by this fact. The climate is also transitional regarding the northern sites being moderately cool, whereas the southern areas—which lay beneath 200 m—are rather moderately warm and dry. Yearly precipitation varies between 400 and 600 mm in the plains, with average annual temperatures over 10 degrees Celsius, whereas the hilly areas are more humid and are characterised by lower yearly average temperature [31]. The site is characterised by chernozem brown forest soil, with sandy loam texture in the upper and sand in the deeper layer.

### 5.2. Experimental Design and Treatments

The measurements took place between 2017 and 2020, following the transformation of a black locust (*Robinia pseudoacacia* L.) energy plantation which had been established for research purposes earlier.

**Figure 6:**
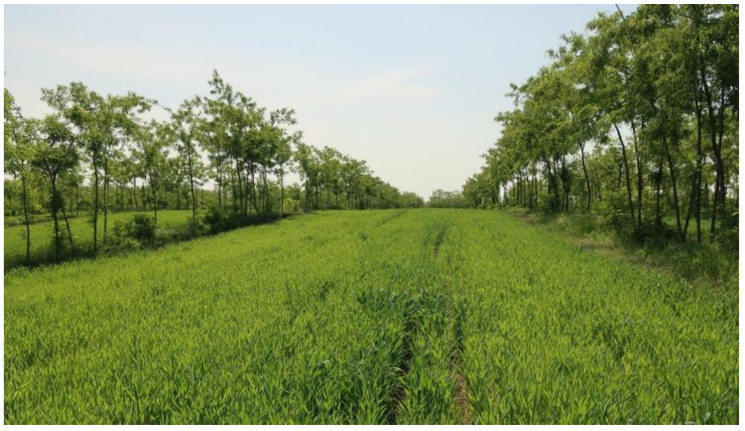
The alley cropping experimental site in Hungary, consisting of black locust (*Robinia pseudoacacia* L.) trees and triticale (x *Triticosecale* W.). (Photo: Veronika Honfy).

Following the transformation to an alley cropping experiment, it became possible to investigate the trees at the ages of three to six and conduct research in the alleys, by intercropping them with triticale (x *Triticosecale* W.), when the trees were 4 and 5 years old (Figure 6). The investigations took place in a near-level plot of 2.3 of which 0.8 hectare was sown in the conventional way of cereal production, and the alley cropping took place in 1.5 hectares. The tree rows were north–south oriented. In 2018 and 2019 the trees were pruned up to two-thirds of their trunk, and the maintenance of the tree rows was conducted twice a year by petrol trimmer.

#### 5.2.1. Black Locust Stands

**Figure 7:**
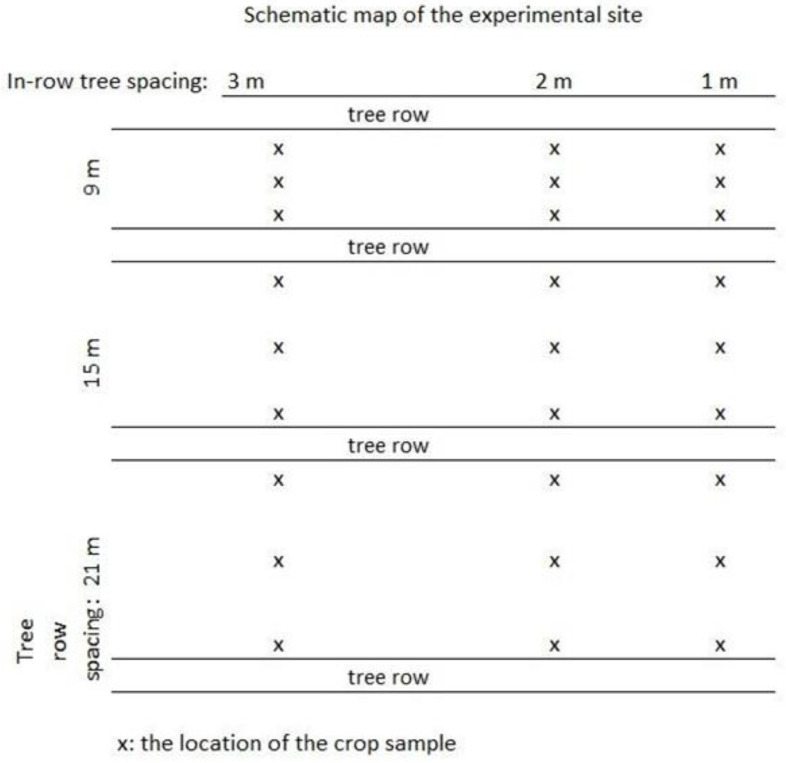
Schematic map of the experiment. Replications are not shown. “x” marks the sampling points of the intercrop.

The energy plantation (short rotation coppice) was established in 2012 of commercial black locust (*Robinia pseudoacacia*), and it was only harvested once, in 2015. At the end of 2016 and beginning of 2017, it was transformed to the alley cropping experiment by sprouting. The original planting spacing for the short rotation coppice was 3.0 × 0.5 m, which was thinned to 9-, 15- and 21-m row spacing, and 1-, 2- and 3-m in-row spacing as shown in Table 6. Thus, the nine different planting spacings are the actual treatments in the experiment. Each tree spacing corresponds to a treatment, namely: 9 × 1, 9 × 2, 9 × 3, 15 × 1, 15 × 2, 15 × 3, 21 × 1, 21 × 2 and 21 × 3. A schematic map of the treatments can be seen in Figure 7.

#### 5.2.2. Triticale

In 2017 and 2018 winter triticale (x *Triticosecale*) was sown in between the tree rows as intercrops, and also in the control area, where there were no trees (due to clearing it); in the previous years, it was also cultivated through black locust energy plantation. Following the removal of tree roots, soil preparation was performed with disks. Maros, a winter variety of triticale, was sown with a 12-cm row spacing. There was no fertiliser or pesticide used in the two years, and only the bare (maintenance) rows near the trees were disked twice a year. The parameters of triticale production are summarised in Table 7.

### 5.3. Sample Collection and Sample Preparation

#### 5.3.1. Dendrometric Measures

Dendrometric measures took place between 2017 and 2020 following the vegetation period of each year, when the trees were 3–6 years old, respectively. In this study we evaluated the results of 2018 and 2019, when the trees were 4 and 5 years old, respectively, because triticale harvests only took place in these years. Electronic tree height measuring equipment (Vertex IV-360) was used to measure height determined in centimetres, and for the diameter at breast height (DBH), an electronic digital calliper (Psion Organizer II) was used, applying two measurements perpendicular to each other. These data were recorded in centimetres with two decimals. All of the trees in the experimental site were measured, which resulted in 489 and 497 records in 2018 and 2019, respectively. Due to the experimental design, each layout (treatment) was only valid in certain tree rows, therefore the dataset had to be filtered according to this. Replications depended on the number of trees recorded in each treatment, which varied between 15 and 38.

#### 5.3.2. Tree Volume and Tree Mass

Tree volume was counted using the formula of Király based on the work of Sopp and Kolozs [32] with the following Equation (1):

(1) $$v=10^{-8}d^{2}h^{1}{\left(h/\left[h-1.3\right]\right)}^{2}\left[-0.6326dh+20.23d\;0.0h+3034\right]$$

where

*v*: tree volume (m^3^);

*d*: diameter at breast height (cm);

*h*: tree height (m).

Above-ground dry dendromass was calculated on the basis of absolute dry bulk density of black locust in Hungary, which is 728 kg m^−3^ [33].

#### 5.3.3. Stand Volume and Above-Ground Dry Dendromass

Stand volume and above-ground dry dendromass were calculated by tree volume or dry mass multiplied by the number of trees per hectare.

#### 5.3.4. Triticale Sample Collection

Triticale samples were collected in July of 2018 and also in 2019. Due to the shading effect of the trees, each plot consisted of three subplots: 3 m away from the tree row to the east and west side of the trees (perpendicular to the tree rows) and one in the middle between two tree rows (Figure 7). In each subplot, all the plants were collected with their roots by hand, with the help of a quadrate of 50 × 50 cm. All of the nine treatments (tree spacing) had four replications, with 3 subplots plus the control plots, which made up 117 subplots in 2018 and 109 in 2019.

#### 5.3.5. Triticale Yields

After collection of plant materials from the 0.25 m^2^ subplots, 10 average plants were selected from each subplot. Following the removal of the spike from the stem and the chaff of the seed, each sample was weighed (Precisa 6200D scale) in grams with one decimal, and the absolute dry matter content was determined with a cereal seed moisture analyser (Pfeuffer HE 50). In each subplot, the number of spikes was also recorded. Based on this, the yield of each m^2^ was calculated by multiplying the absolute dry mass of each “spike” (without the chaff) with the number of spikes in a subplot times four. All yields data represent the absolute dry matter. Yields were determined on a square metre (g m^−2^) and on a hectare basis (t ha^−1^) in 1 hectare of agroforestry, and as relative yields to the control plot.

### 5.4. Defining the Features of Each Treatment

The experimental site was created by transforming an energy plantation into an alley cropping system; therefore, the original planting spacing was defined by 3 × 0.5 m. First, the tree spacings were decided as the multiples of the original layout; then, the rest of the calculations were done, which are listed below.

- Growing space (m^2^): the multiple of the row and in-row spacing of the trees;
- Number of trees (pcs ha^−1^): number of trees taking into account the requirement defined by the Hungarian Law of Forestry, which is to start planting 5 m away from the edge of the field, that would practically result in 8100 m^2^ for tree plantation;
- Width of tree row (m): un-sown strip 0.5 m to the west and 2.5 m to the east side of the trees;
- Length of tree row: based on the same principle as the number of trees, having 90 flow metre for planting, where 0 m and 90 m are the planting points of trees, of which an extra 0.5 m has to be left out of sowing for the protection of the trees (not to be damaged by machinery);
- Number of tree rows: assuming that the length of all sides of the parcel are equal;
- Un-sown area (m^2^): the size of the area excluded from the sowing due to the tree rows;
- Ratio of sown area per hectare (%).

### 5.5. Land Equivalent Ratio

Land equivalent ratio was calculated using the following Equation (2) [34] for all the nine treatments, based on the triticale yields measured in the control plot.

(2) $$LER=L_{A}+L_{B}=\frac{Y_{A}}{S_{A}}+\frac{Y_{B}}{S_{B}}$$

where

*LER*: land equivalent ratio;

*L_A_*, *L_B_*: LER for individual crops;

*Y_A_*, *Y_B_*: individual crop yields in intercropping;

*S_A_*, *S_B_*: yields as sole crops.

There were no control plots for the black locust stands. Therefore, data used for these calculations originated from the literature. The reference data was based on the yield tables of black locust plantations in Hungary, which was published by Rédei [26]. The yield tables are only available from the age of five, therefore the calculations were performed for the year 2019, when the black locust stands were 5 years old in the experiment. Throughout the study best effort was made to give the most precise results, and keeping the overall objective in mind—which was to scale up agroforestry—there was a strong practical approach while defining the features of each treatment, which was the basis of all calculations on yields. Here, we hypothesise that a farmer would implement agroforestry according to local forestry law, and this also led us to be accurate about the size of the sowing area in each treatment. A key difference in alley cropping from pure plantation forestry is that the spaces between the trees can be utilised by the plants, which will then be taken into account when evaluating overall production. When calculating LER, we had to, however, apply the theoretical forestry standard and recount the yields per hectare (of trees and of the crop), to align with the methodology in forestry (theoretical number of trees on 10,000 m^2^) to allow us to count LER of forestry.

### 5.6. Statistical Analysis

Data processing and statistical analyses were conducted using STATISTICA (Version 11) and Microsoft Excel (Office 2019) software. Following descriptive statistics, when the assumption of normality or heterogeneity was not fulfilled, a non-parametric Kruskal–Wallis ANOVA test was performed to see if there were differences between individual trees’ above-ground mass, the total above-ground dendromass per hectare and between the yield of the intercrop according to the treatments. Simple linear regression analyses were performed to test the relationship between stand density and the total volume of stands, and between the yield of the intercrop and number of trees per hectare.

## Figures and Tables

**Table 1:** Features defining each treatment of the alley cropping experiment based on one hectare.

Tree Spacing (m × m)	Growing Space (m^2^)	Number of Trees (pcs ha^−1^)	Width of Tree Rows (m)	Length of Tree Rows (m)	Number of Tree Rows (pcs ha^−1^)	Un-Sown Area (m^2^)	Sown Area (m^2^)	Ratio of Sown Area per Hectare (%)
9 × 3	27	341	3	91	11	3003	6997	70
9 × 2	18	506	3	91	11	3003	6997	70
9 × 1	9	1001	3	91	11	3003	6997	70
15 × 3	45	217	3	91	7	1911	8089	81
15 × 2	30	322	3	91	7	1911	8089	81
15 × 1	15	637	3	91	7	1911	8089	81
21 × 3	63	155	3	91	5	1365	8635	86
21 × 2	42	230	3	91	5	1365	8635	86
21 × 1	21	455	3	91	5	1365	8635	86
Control	-	0	-	-	-	-	10,000	100

Explanation: Number of trees (pcs ha^−1^): number of trees taking into account the requirement defined by the Hungarian Law of Forestry, which is to start planting 5 m away from the edge of a field, that would practically result in 8100 m^2^ for tree plantation. Width of tree row (m): un-sown strip of 0.5 m to the west and 2.5 m to the east side of the trees. Length of tree row: based on the same principle as the number of trees, having 90 m flow for planting, where 0 m and 90 m are planting points of trees, of which an extra 0.5 m has to be left out of sowing, for the protection of the trees (not to be damaged by machinery). Number of tree rows: assuming that the length of all sides of the parcel are equal. Un-sown area (m^2^): the size of the area excluded from the sowing due to the tree rows.

**Table 2:** Volume and absolute dry mass of individual black locust trees at the ages of 4 and 5. The data is ordered by ascending order of growing space (α = 0.05; 2018: n = 272; 2019: n = 263).

Growing Space (m^2^)	Tree Space (m × m)	Average Tree Volume (m^3^)		Average Dry Mass (kg)	
		2018	2019	2018	2019
9	9 × 1	0.017	0.023	12.0 a	16.7 A
15	15 × 1	0.017	0.024	12.2 a b	17.3 A B
18	9 × 2	0.021	0.029	15.2 a b	21.0 A B
21	21 × 1	0.017	0.025	12.4 a b	17.9 A B
27	9 × 3	0.020	0.028	14.6 a b	20.0 A B
30	15 × 2	0.025	0.036	18.0 b	26.2 B
42	21 × 2	0.017	0.023	12.2 a c	16.5 A
45	15 × 3	0.024	0.037	17.7 b c	27.0 B
63	21 × 3	0.017	0.023	12.5 a b	17.0 A

Different letters mark significant differences (α = 0.05) between the treatments in each year.

**Table 3:** Total volume and total above-ground dry dendromass of black locust stands per hectare in alley cropping systems in 2018 and 2019 according to the growing spaces (α = 0.05; 2018: n = 272; 2019: n = 263).

Growing Space (m^2^)	Tree Spacing (m × m)	Total Volume (m^3^ ha^−1^)		Total Dry Dendromass of Stands (atro t ha^−1^)	
		2018	2019	2018	2019
9	9 × 1	16.5	22.9	12.0 a	16.7 A
15	15 × 1	10.7	15.1	7.8 a, b, c	11.0 A, B, C
18	9 × 2	10.6	14.6	7.7 a, b	10.7 A, B
21	21 × 1	7.8	11.2	5.7 b, c	8.2 B, C
27	9 × 3	6.9	9.4	5.0 c	6.8 C
30	15 × 2	8.0	11.6	5.8 b, c	8.4 A, C
42	21 × 2	3.9	5.2	2.8 d	3.8 D, E
45	15 × 3	5.3	8.1	3.8 c, d	5.9 C, D
63	21 × 3	2.7	3.6	1.9 d	2.6 E

Different letters mark significant differences (α = 0.05) between the treatments in each year.

**Table 4:** Yields of triticale according to different layout of alley cropping systems and at the control plot (without trees). The trees were 4 and 5 years old. (α = 0.05; 2018: n = 775, 2019: n = 1031).

Tree Spacing (m × m)	Number of Trees (pcs ha^−1^)	Sown Area (m^2^)	Mean DM Yield (g m^−2^)		Stand. Dev. Of Mean DM Yield (g m^−2^)		Mean DM Yield (t ha^−1^)		Stand. Dev. of Mean DM Yield (t ha^−1^)	
			2018	2019	2018	2019	2018	2019	2018	2019
9 × 1	1001	6997	274.4 *	150.8 E	122.2	60.74	1.92 *	1.06	0.9	0.4
9 × 2	506	6997	407.8 *	173.2 B E	222.0	95.2	2.85 *	1.21	1.6	0.7
9 × 3	341	6997	272.2 *	110.9 F	165.2	58.3	1.90 *	0.78	1.2	0.4
15 × 1	637	8089	297.2 a c	172.8 B E	110.6	80.7	2.40	1.40	0.9	0.7
15 × 2	322	8089	425.8 b	262.1 A	147.3	104.5	3.44	2.12	1.2	0.8
15 × 3	217	8089	331.2 a c	244.6 A B	142.3	156.5	2.68	1.98	1.2	1.3
21 × 1	455	8635	352.2 a d	232.2 A C	167.8	121.6	3.04	2.01	1.4	1.1
21 × 2	230	8635	392.4 b d	189.5 B C E	141.1	100.2	3.39	1.64	1.2	0.9
21 × 3	155	8635	280.9 c	206.0 B C D	83.4	113.1	2.43	1.78	0.7	1.0
Control	0	10,000	335.1 a c	192.6 A D E	120.3	95.3	3.35	1.93	1.2	1.0

Different letters show significant differences between the treatments. * In 2018, in the treatments of 9 m row space the sowing rate was 220 kg ha^−1^, whereas the rest of the treatments were sown with a sowing rate of 180 kg. In 2019, sowing rate for all treatments was 220 kg ha^−1^. There was no fertilisation in any of the years, which could explain the great reduction of yields in 2019.

**Table 5:** Land equivalent ratios (LER) for each tree spacing.

Tree Spacing	LER_crop_	LER_forest_	LER_agroforestry_
15 × 2	1.08	0.27	1.35
21 × 1	1.03	0.26	1.29
15 × 3	1.01	0.18	1.19
9 × 1	0.50	0.57	1.07
15 × 1	0.71	0.36	1.07
21 × 3	0.91	0.08	0.99
21 × 2	0.84	0.12	0.96
9 × 2	0.58	0.36	0.94
9 × 3	0.37	0.27	0.64

**Table 6:** The treatments of the alley cropping experiment, which are actually the different growing spacings of black locust stands. In brackets, the growing spacings are shown for each tree.

	Tree in-Row Spacing (m)			Control
Tree Row Spacing (m)	1	2	3	No Trees
9	9 × 1 (9 m^2^)	9 × 2 (18 m^2^)	9 × 3 (27 m^2^)	FS (Full Sun)
15	15 × 1 (15 m^2^)	15 × 2 (30 m^2^)	15 × 3 (45 m^2^)	
21	21 × 1 (21 m^2^)	21 × 2 (42 m^2^)	21 × 3 (63 m^2^)	

**Table 7:** Growing technology of triticale of the harvesting years 2018 and 2019.

	2018	2019
Date of sowing	20 October 2017	10 October 2018
Sowing rate	220 kg ha^−1^ at 9 m row space treatments 180 kg ha^−1^ at the rest of the treatments	220 kg ha^−1^
Fertilization	not applicable	not applicable
Plant protection	not applicable	not applicable
Date of harvest	9–12 July 2018	8–11 July 2019

## Data Availability

Not applicable.
